# Hopelessness and burnout in Italian healthcare workers during COVID-19 pandemic: the mediating role of trait emotional intelligence

**DOI:** 10.3389/fpsyg.2023.1146408

**Published:** 2023-05-05

**Authors:** Maria Stella Epifanio, Sabina La Grutta, Marco Andrea Piombo, Martina Riolo, Vittoria Spicuzza, Marianna Franco, Giacomo Mancini, Leonardo De Pascalis, Elena Trombini, Federica Andrei

**Affiliations:** ^1^Department of Psychology, Educational Science and Human Movement, University of Palermo, Palermo, Italy; ^2^Department of Psychology “Renzo Canestrari,” Alma Mater Studiorum - University of Bologna, Bologna, Italy; ^3^Department of Education Studies “Giovanni Maria Bertin,” Alma Mater Studiorum - University of Bologna, Bologna, Italy; ^4^Psychological Sciences, Institute of Health and Life Sciences, University of Liverpool, Liverpool, United Kingdom

**Keywords:** COVID-19, pandemic, healthcare workers, burnout, hopelessness, trait emotional intelligence, TEIQue-SF

## Abstract

**Objective:**

The study aims to assess the impact of COVID-19 on healthcare workers’ work-related stress during the first wave of the pandemic in Italy. The main objective is to investigate the existence of a positive correlation between hopelessness and burnout, assuming that burnout may be a risk factor for the development of hopelessness, and to analyze the role that trait Emotional Intelligence (TEI) and changes in workload could have in this relationship. Furthermore, evaluate any significant differences in burnout and hopelessness levels in the function of some demographic variables, such as gender, professional profiles, and different working zones of Italy, to better understand how the diverse diffusion of pandemic had affected Italian healthcare workers.

**Methods:**

An online survey was used to collect data between April and June, 2020, with 562 responses among nurses (52.1%) and physicians (47.9%). Demographics and changes in workload and work conditions were collected through an *ad hoc* questionnaire. The Trait Emotional Intelligence Questionnaire-Short Form (TEIQue-SF), The Beck Hopelessness Scale (BHS), and the Link Burnout Questionnaire (LBQ) were used to assess Trait Emotional Intelligence (TEI), hopelessness, and burnout, respectively.

**Results:**

Correlation analysis showed a significant positive relationship between hopelessness and each burnout dimension. TEI showed negative correlations both with burnout dimensions and hopelessness. Significant differences in burnout and hopelessness levels emerged as a function of some demographic variables such as gender, professional profiles (nurses or physicians), and different working zone of Italy (northern or southern). Results showed that TEI partially mediated the relationship between hopelessness and every burnout dimension, while the interaction of changes in workload was non-significant.

**Discussion:**

The mediating role of TEI in the burnout-hopelessness relationship partly explains the protective role that individual factors had on healthcare workers’ mental health. Our findings support the need to integrate considerations on both psychological risk and protective factors into COVID-19 care, including the monitoring of psychological symptoms and social needs, especially among healthcare workers.

## Introduction

### Background

The COVID-19 pandemic has been deeply affecting both the general population and the health systems of different countries. Several international studies have shown an increase in mental suffering (Rossi et al., 2020; Zhang et al., 2020; Breslau et al., 2021; Lorant et al., 2021; Daly and Robinson, 2022) and the worsening of quality of life (QoL; Horesh et al., 2020; White and Van Der Boor, 2020; Epifanio et al., 2021; Van Ballegooijen et al., 2021; Hansel et al., 2022) especially on healthcare professionals (Bellizzi et al., 2020; Guanche Garcell, 2020; Semple and Cherrie, 2020; Cortés-Álvarez and Vuelvas-Olmos, 2022).

During the first pandemic wave, Italy was among the most affected nations in terms of hospital overload, and its healthcare workforce struggled to face all the challenges related to the pandemic, especially in the northern regions of the country. According to the integrated surveillance report on COVID-19 in Italy^1^ in November 2020, 446.875 health workers were infected,^2^ and there were 378 deaths among doctors.

In this context, it has become necessary to understand the COVID-19 epidemic health consequences on Italian health professionals at the forefront (Guanche Garcell, 2020). Several studies on the impact of COVID-19 on healthcare workers’ mental health (Moitra et al., 2021; Saragih et al., 2021; Sun et al., 2021; Dragioti et al., 2022) found a high prevalence of post-traumatic stress disorder, depressive and anxious symptoms, sleep disorders, psychological distress, and burnout syndrome.

Stress is caused not only by the continuous contact with the patient’s suffering but also by other factors related to the workplace, such as its organization, dynamics, and quality of relations with colleagues and health management (Romano, 2005; Schaufeli et al., 2009).

Recurrence of distress situations could lead to a high burnout risk, a particular type of acute and extreme physical and psychological suffering (Maslach and Jackson, 1981; Maslach and Schaufeli, 1993; Maslach, 2003) resulting from the perception of an imbalance between the number of requests and the resources available, in order to positively respond to such requests. This can lead to energies depletion, with subsequent physical symptoms (frequent headaches, insomnia, drug use), psychological symptoms (guilt, negativism, irritability), behavioral reactions, and absence and delay at work (tendency to avoid telephone contact, changes in attitude, emotional detachment, and indifference to patient’s problems; Barello et al., 2020; Kang et al., 2020; Lai et al., 2020). Early research on burnout (Maslach and Schaufeli, 1993) suggests that it can lead to a deterioration of care or service quality provided by medical professionals. In fact, operator feelings can include tension, irritability, and emotional excitability that could lead to detachment from their users in order to preserve their energies and resources.

In addition, burnout can be characterized by a motivation decline that leads to a loss of idealism, enthusiasm, positivity, and increases in apathy and cynicism toward users (Maslach, 2003). It is clear from this that personal satisfaction and gratification, together with empathy toward their users, are essential to provide effective care. Therefore, healthcare professionals’ burnout has negative consequences for staff, patients, and organizations, particularly in the case of frontline workers (Friedberg et al., 2014). For instance, burnout compromises the quality of care due to turnover (Leiter et al., 1998), absenteeism (Davey et al., 2009), and early withdrawal (Linzer et al., 2001). The clinical and economic impact of burnout is such that in 2019, it was included in the 11th revision of the International Classification of Disease (World Health Organization, 2022) as an employment phenomenon. According to the definition of ICD-11, burnout is a syndrome resulting from chronic stress in the workplace, not properly managed. It is characterized by a feeling of depletion of energy or exhaustion, an increase in mental distance and negative or cynical feelings toward work and others, and reduced professional effectiveness.

In their efforts to assist and treat patients with COVID-19, physicians and nurses in particular (Liu et al., 2020), have been exposed to daily events that may lead to the development, or exacerbation, of psychological distress. Moreover, the very long work shifts, the constant fear of contagion, and the frustration caused by the difficulty in providing effective care are all risk factors that may have contributed to the onset of considerable work distress (Lai et al., 2020; Baptista et al., 2021).

Several studies (Franza et al., 2020; Martínez-López et al., 2020; Morgantini et al., 2020; Rossi et al., 2020; Ghahramani et al., 2021; Gualano et al., 2021; Claponea et al., 2022; Kimpe et al., 2022) have shown a high prevalence of burnout syndrome in healthcare workers during COVID-19 pandemic. Zhang et al. (2020) found a 56.03% prevalence of anxiety symptoms and burnout in the Intensive Care Unit (ICU) medical staff. Other studies (Dinibutun, 2020; Alrawashdeh et al., 2021; Baptista et al., 2021; Tuna and Özdin, 2021) show similar results both in family doctors (Baptista et al., 2021) and in frontline physicians (Tuna and Özdin, 2021).

Regarding nurses, international studies report around an 11% prevalence of burnout worldwide (Woo et al., 2020; Galanis et al., 2021; Zareei et al., 2022). In other words, one-tenth of the world’s nurses show symptoms of burnout syndrome. Moreover, among gender-related burnout, data in the literature (Innstrand et al., 2011; Vitale et al., 2020) suggest that females tend to experience higher levels of psychophysical exhaustion probably due to women’s double workload and inequality between genders at work.

There are many risk factors for burnout at both individual and institutional levels (Bria et al., 2012). In a systematic review, Bria et al. (2012) identified some risk factors at the institutional level, such as perceived job control, high workload, organizational justice, social support at work, effort-reward imbalance, perceived burnout complaints among colleagues, and hospital organizational characteristics. Moreover, a recent review by Khosravi et al. (2022) on healthcare staff’s burnout during the COVID-19 pandemic highlights the key role of environmental factors, such as role ambiguity, role conflict, and work overload, in increasing the severity of burnout (Khosravi et al., 2022) and reports several contributions from the literature on possible strategies for treating and preventing burnout among healthcare workers, such as interventions focused on increasing the awareness of occupational burnout (Maldonato et al., 2020) or interventions based on the promotion of supportive work culture in order to elevate the healthcare workers’ resilience (Dewey et al., 2020).

Concerning individual risk factors, studies (Demir et al., 2003; Sharma et al., 2008) show that healthcare professionals who experienced burnout use more emotion-focused coping such as substance abuse and unhealthy eating habits. In healthcare workers, hope is one of the main coping strategies that influence the ability to interact with stress in life-threatening situations (Franza et al., 2020). Hopelessness, in fact, leads to the absence of determination and catastrophic and destructive thoughts, and it is well-documented that physicians take their own lives at rates much higher than the general public, and on average, 400 US physicians die by suicide each year (Bradley and Chahar, 2020). In Italy (Lorettu et al., 2020) it seems that there are more than 25 suicide every year among physicians, while data about nurses are not available.

One of the most important risk factors for suicide is certainly hopelessness, a psychological construct (Beck et al., 1974) that has been identified as one of the characteristics of depression and has been implicated in a variety of other conditions such as schizophrenia and alcoholism. The few studies that investigated the relationship between hopelessness and burnout (Pompili et al., 2013; Franza et al., 2020; Karagol and Kaya, 2022) found a positive correlation between the two constructs, assuming that burnout may be a risk factor for the development of hopelessness, which could lead to the onset of depression and suicide risk.

In contrast to these risk factors, there are personal dispositions that could be important in preventing burnout and hopelessness, such as Emotional Intelligence (EI). EI is generally defined as a psychological attribute that captures individual differences in how we perceive, communicate, regulate, and understand our own emotions, as well as the emotions of others (Hughes and Evans, 2018). The ability to monitor one’s own and others’ feelings and emotions and to guide one’s thinking and behavior seems to be a useful competency in dealing with stress work-related (Humpel and Caputi, 2001). In line with other studies (Humpel and Caputi, 2001; Swami et al., 2013; De Moraes et al., 2015). Adamson (2014) found that nurses with high EI were less likely to experience burnout than those with low scores. Moreover, other studies found a negative relationship between EI and burnout (Mérida-López and Extremera, 2017; Fiorilli et al., 2019; Tesi, 2021).

Among the different possible EI formulations, some theories see EI as a factor that promotes general health (Schutte et al., 2007; Zysberg, 2018) and better adaptation (Pellitteri, 2002) influencing the way individuals cope with demands and pressures from the environment to be more resilient to challenging situations (Zeidner et al., 2006) and uses specific tests to measure emotional abilities. Trait emotional intelligence (TEI), instead, focuses on people’s perception of their emotional disposition and is identified as a distinct latent variable that integrates the affective aspects of personality (Andrei et al., 2014, 2015). Particularly, the construct of TEI has emerged as an important individual difference variable referring to a constellation of emotional self-perceptions assessed by self-reported questionnaires (Petrides et al., 2007). Several studies have explored the influence of TEI across the lifespan (Petrides et al., 2016) and its impact on health, underlining that high TEI strongly positively predicts wellbeing, mental health (e.g., Martins et al., 2010), and moderates the responses to stress (Mikolajczak and Luminet, 2008). In this sense, this type of framework and measurement are best suited to the purposes of our research, which assesses how personal disposition (e.g., the tendency toward hopelessness) and situational (the ongoing pandemic and consequent changes in the burden of work) affect work-related stress. To our knowledge, while recent studies examined the role of trait EI in the relationship between several stressful experiences, such as job loss during the COVID-19 Pandemic (Andrei et al., 2022), few studies explore the relationship between trait EI, burnout, and hopelessness in a high-risk sample such as healthcare workers, especially those who were in the frontline during COVID-19 Pandemic.

### The present study

The study aims to assess the impact of COVID-19 on work-related stress and risks for healthcare workers’ mental health during the first wave of the pandemic in Italy. The main objective was to investigate the existence of a positive correlation between hopelessness and burnout, assuming that burnout may be a risk factor for the development of hopelessness, and to analyze the role that trait EI and changes in workload due to the pandemic situation could have in this relationship. Another aim of this study was to evaluate the differences in burnout and hopelessness levels taking into account gender, professional profiles (nurses or physicians), and different working zone of Italy (northern or southern).

We hypothesized that: (1) healthcare workers who work in a COVID-19 ward and northern regions of Italy have higher levels of burnout and hopelessness than others due to the different diffusion that the pandemic had in the first wave; (2) nurses have higher levels of burnout and hopelessness than physicians due to their first-responder role; (3) females have a higher level of psychophysical exhaustion burnout dimension, as confirmed by previous literature, due to women’s double workload and inequality between men and women in work contexts; (4) the existence of a positive correlation between hopelessness and burnout, assuming that burnout may be a risk factor for the development of hopelessness; (5) trait EI (TEI) will mediate the relationship between burnout and hopelessness, while changes in workload due to the pandemic situation could be a moderator factor of this relationship; and (6) higher workload could cause more burnout, consequentially increasing the feeling of hopelessness.

## Materials and methods

### Procedures

An online cross-sectional survey was performed with Qualtrics® (Qualtrics, Provo, UT, United States) Survey Platform. This data collection strategy was chosen as it allowed us to reach as many voluntary participants as possible in a phase of forced social distancing. The survey started after 7 weeks of quarantine in Italy (25 April 2020) and was performed for about 6 weeks, until the end of lockdown measures (2 June 2020). The sample was recruited via a snowball sampling strategy. A link to Qualtrics questionnaires were sent via e-mail, social networks (Facebook and WhatsApp), and official working platforms (website of the University of Palermo, Italy).

A brief presentation informed the participants about the aims of the study, and electronic informed consent, assuring maximum confidentiality in the handling and analysis of the responses, was requested from each participant before starting the investigation. The survey took approximately 30 min to complete. Participation was voluntary and free of charge. To guarantee anonymity, no personal data, which could allow the identification of participants, were collected.

Participants could withdraw from the study at any time without providing any justification, and the data were not saved. Only the questionnaire data with a complete set of answers by respondents were considered. The study was conducted in accordance with the Declaration of Helsinki and was approved by the Bioethics Committee of the University of Palermo (n. 7/2020).

### Participants

Italian healthcare workers who were working in public hospital wards COVID-19 and NO-COVID-19 and outpatient clinics at the time of the first wave pandemic (April–July 2020) were eligible for participation to data collection. The recruited sample size through the online survey included 774 Italian adults, with an attrition rate of approximately 27%. Our final sample comprised 562 respondents, physicians (*n* = 269) and nurses (*n* = 293) Demographic characteristics of the study sample are presented in Table 1.

**Table 1:** Mean ± standard deviation for main study variables divided by gender, region, professional profile, and COVID-19 ward.

		Gender	Region		Professional profiles		COVID wards	
	Female	Male	North	South	Nurses	Physicians	Yes	No
PHE	20.77 ± 6.50	18.77 ± 6.63^**^	20.60 ± 6.35	18.66 ± 7.23^**^	20.08 ± 6.72	20.35 ± 6.45	20.40 ± 6.55	19.82 ± 6.66
DET	16.94 ± 5.20	16.28 ± 4.8	16.90 ± 4.83	16.22 ± 5.97	17.00 ± 5.24	16.49 ± 4.92	16.96 ± 5.03	16.35 ± 5.21
JIN	14.62 ± 4.90	13.37 ± 5.05^**^	14.37 ± 4.76	13.88 ± 5.67	14.08 ± 4.83	14.48 ± 5.11	14.29 ± 5.080	14.23 ± 4.75
DIS	15.82 ± 7.37	15.37 ± 7.09	16.02 ± 7.13^*^	14.42 ± 7.763	15.92 ± 7.52	15.45 ± 7.04	15.63 ± 7.42	15.82 ± 7.05
WOR	0.41 ± 0.71	0.26 ± 0.79^*^	0.43 ± 0.711	0.14 ± 0.79^**^	0.49 ± 0.67	0.24 ± 0.78^**^	0.48 ± 0.69	0.15 ± 0.77^***^
TEI	4.96 ± 0.70	5.11 ± 0.76^*^	4.97 ± 0.70	5.12 ± 0.78^*^	4.92 ± 0.71	5.09 ± 0.72^**^	4.98 ± 0.72	5.04 ± 0.71
HPS	6.93 ± 0.4.98	6.80 ± 0.5.08	7.00 ± 0.4.92	6.53 ± 0.5.29	6.61 ± 4.95	7.21 ± 5.05	6.99 ± 0.5.02	6.70 ± 4.991

PHE, Psychophysical Exhaustion; DET, Deterioration of relations with clients; JIN, Job Ineffectiveness; DIS, Disappointment; WOR, Workload; TEI, trait Emotional Intelligence; HPS, Hopelessness. ^*^p < 0.05, ^**^p < 0.01, ^***^p < 0.001.

### Measures

#### Demographics and changes in workload information questionnaire

An *ad hoc* questionnaire was created to collect demographic data (such as gender, age, region of residence in Italy, job position) and work information (i.e., changes in employment status, workload, years of professional experience, public hospital, COVID-19 and NO-COVID-19 wards, and public out-patient clinic).

#### The Trait Emotional Intelligence Questionnaire-Short Form

The Trait Emotional Intelligence Questionnaire–Short Form (TEIQue-SF; Petrides, 2009; Di Fabio and Palazzeschi, 2011) was used to assess trait EI. Consist of 30 items rated on7-point Likert scale, provides a global trait EI score and scores at four factors (i.e., wellbeing, self-control, emotionality, and sociability), which higher scores corresponds to high levels of trait EI. Reliability for the present study was good (Cronbach’s alpha = 0.90).

#### The Beck Hopelessness Scale

The Beck Hopelessness Scale (BHS; Beck et al., 1974; Pompili et al., 2013) was used to measure feeling of hopelessness, BHS includes 20 true/false items about the future (9 positives and 11 negatives), that can be summed up to give a total score ranging from 0 to 20. Higher scores reflect increased hopelessness. In this study, this questionnaire showed good reliability (Cronbach’s alpha = 0.89).

#### The Link Burnout Questionnaire

The Link Burnout Questionnaire (LBQ; Santinello and Altonoè 2007) was used to measure professional burnout, LBQ includes 24 items rated on a 6-point Likert scale. A total score of LBQ allows measuring 4 dimensions of professional burnout: psychophysical exhaustion (dimension referring to the self-assessment of one’s own psychophysical resources); deterioration of relations with clients (dimension referring to the quality of those relations); job ineffectiveness (dimension referring to the self-assessment of one’s own professional competences); and disappointment (dimension of existential expectations). For each dimension, cut-off scores change differently for physicians and nurses: psychophysical exhaustion (low: 6–13 physicians, 6–12 nurses; average: 14–20 physicians, 13–19 nurses; moderate: 21–26 physicians, 20–25 nurses; high: 27–36 physicians, 26–36 nurses); deterioration of relations with clients (low: 6–9 physicians, 6–8 nurses; average: 10–13 physicians, 9–13 nurses; moderate: 14–21 physicians, 14–20 nurses; high: 22–36 physicians, 21–36 nurses); job ineffectiveness (low: 6–10 physicians, 6–10 nurses; average: 11–17 physicians, 11–17 nurses; moderate: 18–23 physicians, 18–24 nurses; high: 24–36 physicians, 25–36 nurses); and disappointment (low: 6–9 physicians, 6–9 nurses; average: 10–16 physicians, 10–16 nurses; moderate: 17–24 physicians, 17–25 nurses; high: 25–36 physicians, 26–36 nurses). Reliability for the present study was good (Cronbach’s alpha = 0.91).

### Data analysis

**Figure 1:**
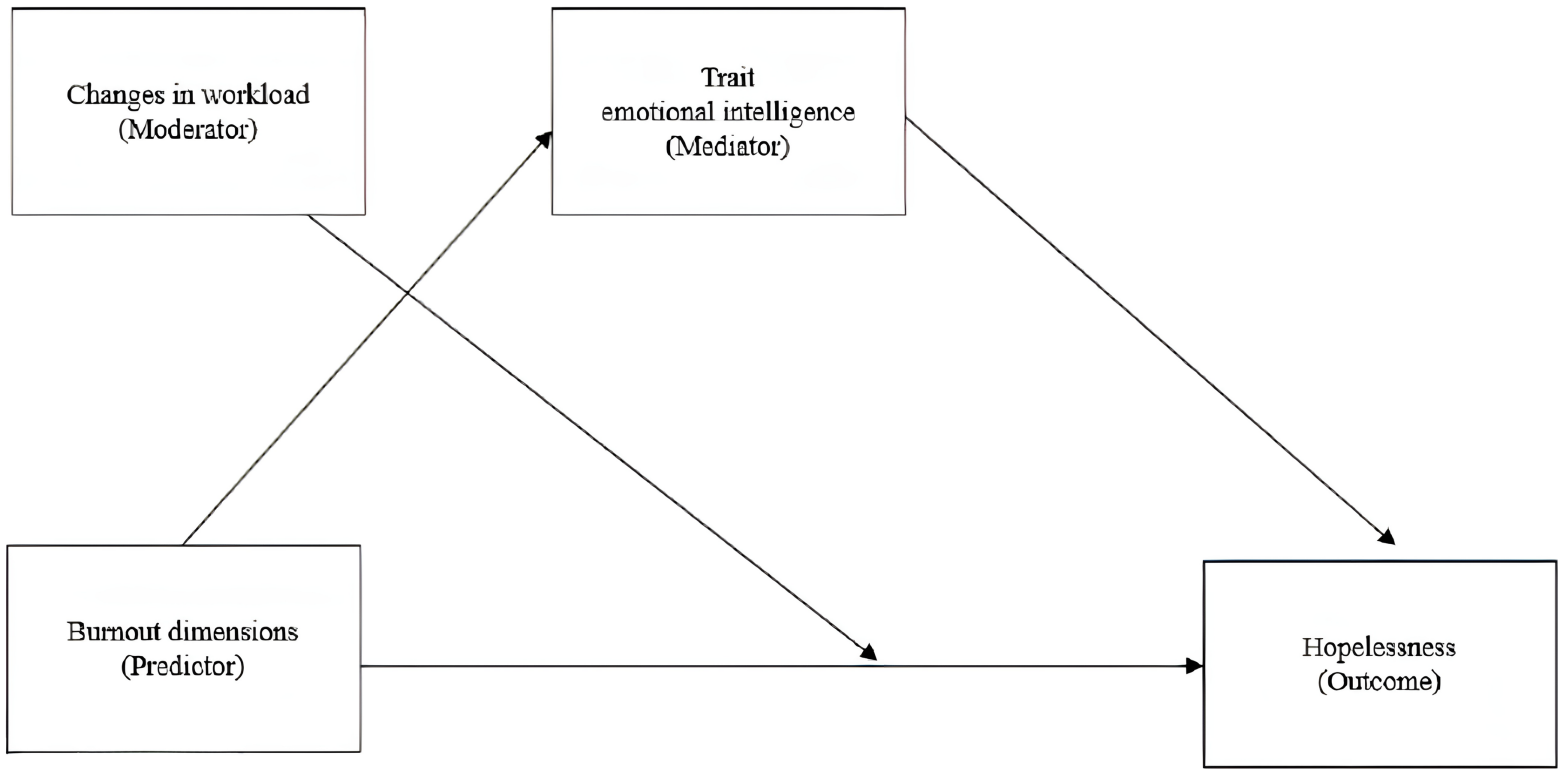
Mediation and moderation model of association between all burnout dimensions, trait emotional intelligence, changes in workload and hopelessness.

Statistical analyses were performed using programs available in the Statistical Package for Social Sciences (SPSS for Windows release 25.0). Descriptive statistics were utilized to describe the data (frequencies, percentages, mean, and standard deviation). Pearson’s correlations were used to investigate associations among variables. Change in workload was coded as a dummy variable: −1 = less workload; 0 = same workload; 1 = more workload than pre-pandemic period. Moreover, also gender (0 = females;1 = males), region (0 = south and 1 = north) and COVID-19ward (0 = No; 1 = Yes) were coded as dummy variables. Multivariate measures of ANOVA were used to investigate differences between groups. Finally, to explain more in-depth the relationship between burnout and hopelessness and to analyze the effect that changes in workload and TEI have on this relationship, some mediation and moderation models were performed with the computational tool for SPSS, PROCESS (Model 5). In particular, every burnout dimension was included in the model as a predictor, while hopelessness was included as a dependent variable. Moreover, changes in workload were included as a moderator factor of this relationship, while TEI was included as a mediator (Figure 1).

## Results

### Descriptive statistics

The final sample comprised a total of 562 healthcare workers (72.2% females), 52.15% nurses, and 47.9% physicians, collected mainly from the northern (78.3%) and southern (21.7%) regions of Italy and from hospitals (75.1%) and outpatient clinics (24.9%). Respondents were mostly middle (age 35–54; 51.6%) and older adults (age ≥ 55; 28.3%), while the group of young adults was smaller (age ≤ 34; 20.1%). Among years of professional experience, most of them had more than 20 years of experience (46.3%), while ≤10 and 11–20 range were, respectively, 28.8 and 24.9%.

Most participants worked in COVID-19 wards at the time of the survey (66.5%), while only 33.1% were in the other wards. Concerning the change in workload, 52% of participants experienced a greater workload, while 33.1% were the same and only 15.7% had a lower workload. Finally, regarding COVID-19 contagion, only 7.3% of participants had personally contracted COVID-19, while 17.8% had relatives who contracted the virus and, among them, 20% died.

### Gender, regions, and working wards comparisons

Table 1 presents results from multivariate ANOVAs. Results showed significant gender differences in two burnout dimensions, with females scoring higher in psychophysical exhaustion (*F* = 10.51; *p* < 0.001) and job ineffectiveness (*F* = 7.23; *p* < 0.01) than males so as in workload changes (*F* = 5.13; *p* < 0.05). Contrary, males score higher than females in TEI (*F* = 4.55; *p* < 0.05). As regards regions of Italy, healthcare workers from North reported significantly higher workload changes (*F* = 19.87; *p* < 0.05) and in two burnout dimensions: psychophysical exhaustion (*F* = 7.89; *p* < 0.01) and disappointment (*F* = 4.37; *p* < 0.05). Finally, regarding professional profiles, nurses reported significantly higher workload changes than physicians (*F* = 15.97; *p* < 0.001) so as those who work in COVID wards (*F* = 25.71; *p* < 0.001). Finally, no differences between groups were found in Hopelessness (all *ps* > 0.05).

### Correlational analyses

Table 2 presents Pearson’s correlation coefficients of study variables. Specifically, hopelessness showed positive correlations with every burnout dimension (psychophysical exhaustion *r* = 0.57, *p* < 0.001; deterioration of relations with clients *r* = 0.35, *p* < 0.01; job ineffectiveness *r* = 0.46, *p* < 0.01; Disappointment *r* = 0.63, *p* < 0.01); and negative correlations with TEI (*r* = −0.59, *p* < 0.01), while TEI showed negative correlation with every burnout dimensions (psychophysical exhaustion *r* = −0.55, *p* < 0.001; deterioration of relations with clients *r* = −0.42, *p* < 0.01; job ineffectiveness *r* = −0.55, *p* < 0.01; and disappointment *r* = −0.56, *p* < 0.01). Finally, Change in Workload showed a significant relationship only with two burnout dimensions: psychophysical exhaustion (*r* = 0.17, *p* < 0.01) and deterioration of relation with Clients (*r* = 0.13, *p* < 0.01).

**Table 2:** Correlational analysis.

	2	3	4	5	6	7	8	9
1. Gender	0.13^**^	**−0.09** ^*^	−0.13^**^	−0.05	**−0.11** ^**^	−0.02	0.09^*^	−0.01
2. Region		**−0.16** ^**^	**−0.11** ^**^	−0.03	−0.02	**−0.08** ^*^	**0.09** ^*^	−0.03
3. Workload			**0.17** ^**^	0.13^**^	0.04	0.02	−0.01	0.07
4. PE				0.55^**^	**0.62** ^**^	**0.71** ^**^	**−0.55** ^**^	**0.57** ^**^
5. DET					**0.50** ^**^	**0.52** ^**^	**−0.42** ^**^	**0.35** ^**^
6. JIN						**0.59** ^**^	**−0.55** ^**^	**0.46** ^**^
7. DIS							**−0.56** ^**^	**0.63** ^**^
8. TEI								**−0.59** ^**^
9. HPS								

PE, Psychophysical Exhaustion; DET, Deterioration of relations with clients; JIN, Job Ineffectiveness; DIS, Disappointment; TEI, trait Emotional Intelligence; HPS, Hopelessness. ^*^p < 0.05, ^**^p < 0.01.

### Differences in burnout levels and hopelessness

Table 3 presents data on burnout dimension levels. Most participants obtained an average score in the dimension of psychophysical exhaustion (41.8%), while 20.6% obtained high, 29% moderate and 8.5% low scores, respectively. Regarding the dimension deterioration of the relationship with customers, 48.6% of the participants obtained a moderate score, while 20.6% high, 29.7% average, and only the 1.1% low scores. Levels of the dimension labeled as work ineffectiveness, scores were high for 5% of participants, moderate for 16.9%, medium for 66.5%, and low for 11.6%. Disappointment subscale scores were high for 13.2% of participants, moderate for 26.9%, medium for 50.7%, and low for 9.6%. Furthermore, women showed higher levels than men in two dimensions of burnout: psychophysical exhaustion (χ^2^ = 17.59; *p* < 0.01) and work ineffectiveness (χ^2^ = 29.04; *p* < 0.01). Nurses, on the other hand, showed higher levels than physicians in the three dimensions of burnout (psychophysical exhaustion: χ^2^ = 8.340; *p* < 0.05; deterioration of the relationship with clients: χ^2^ = 10.87; *p* < 0.05; work Ineffectiveness: χ^2^ = 9.67; *p* < 0.05). Regarding region differences, participants from North Italy showed more high or moderate levels than participants from South in three burnout dimensions (psychophysical exhaustion: χ^2^ = 8.340; *p* < 0.05; deterioration of the relationship with clients: χ^2^ = 10.87; *p* < 0.05; disappointment: χ^2^ = 13.50; *p* < 0.05). Finally, regarding comparisons working with COVID-19 patients or not, participants who worked in COVID-19 wards showed more high or moderate scores only in job ineffectiveness (χ^2^ = 8.00; *p* < 0.05). Contrary, participants who worked in COVID-19 wards showed more low hopelessness levels than who worked in other wards (χ^2^ = 11.07; *p* < 0.05; see Table 3).

**Table 3:** Frequencies of burnout levels, hopelessness, and change in workload.

		Region		Gender		Professional profiles		COVID-19 wards	
		North	South	Female	Male	Nurses	Phys.	Yes	No
PE	Average	41.6%	42.5%	39.7%	47.4%	43.7%	39.8%	41.9%	41.8%
	High	20.7%	20.4%	22.1%	16.7%	23.5%	17.5%	19.9%	21.0%
	Low	5.9%	19.4%	6.2%	14.7%	6.1%	11.2%	11.8%	6.9%
	Moderate	31.8%	17.7%	32.0%	21.2%	26.6%	31.6%	26.3%	30.3%
Chi-squared test	χ^2^	22.00^**^		17.60^**^		8.34^*^		4.27	
DET	Average	27.7%	36.9%	28.1%	34.0%	27.3%	32.3%	33.9%	27.7%
	High	20.2%	22.1%	21.9%	17.3%	25.3%	15.6%	18.8%	21.5%
	Low	0.7%	2.5%	1.2%	0.6%	1.7%	0.4%	1.1%	1.1%
	Moderate	51.4%	38.5%	48.8%	48.1%	45.7%	51.7%	46.2%	49.7%
Chi-squared test	χ^2^	8.93^*^		2.86		10.87^*^		2.38	
JIN	Average	70.7%	51.6%	70.2%	57.1%	71.7%	61.0%	62.4%	68.6%
	High	5.0%	4.9%	5.7%	3.2%	4.1%	5.9%	2.7%	6.1%
	Low	8.4%	23.0%	7.1%	23.1%	8.2%	15.2%	13.4%	10.6%
	Moderate	15.9%	20.5%	17.0%	16.7%	16.0%	17.8%	21.5%	14.6%
Chi-squared test	χ^2^	29.86^**^		29.04^***^		9.68^*^		7.99^*^	
DIS	Average	50.7%	50.8%	51.0%	50.0%	49.8%	51.7%	48.4%	51.9%
	High	12.5%	15.6%	13.8%	11.5%	13.3%	13.0%	12.4%	13.6%
	Low	7.5%	17.2%	8.6%	12.2%	9.9%	9.3%	9.1%	9.8%
	Moderate	29.3%	16.4%	26.6%	26.3%	27.0%	26.0%	30.1%	24.7%
Chi-squared test	χ^2^	16.08**		1.95		0.20		1.85	
HPS	High	10.2%	9.8%	10.1%	10.3%	9.2%	11.2%	7.5%	11.4%
	Low	35.7%	27.9%	34.5%	32.7%	34.1%	33.8%	26.9%	37.5%
	Moderate	23.6%	21.3%	22.7%	24.4%	21.5%	24.9%	28.5%	20.5%
	Normal	30.5%	41.0%	32.8%	32.7%	35.2%	30.1%	37.1%	30.6%
Chi-squared test	χ^2^	5.19		0.25		2.31		11.07^*^	
WOR	Less	13.0%	25.6%	13.3%	21.9%	10.2%	21.6%	23.7%	11.7%
	Same	30.7%	34.7%	32.0%	30.3%	30.7%	32.5%	37.6%	28.5%
	More	56.3%	39.7%	54.7%	47.7%	59.0%	45.9%	38.7%	59.7%
Chi-squared test	χ^2^	15.20^**^		6.45^*^		16.32^**^		24.95^***^	

PE, Psychophysical Exhaustion; DET, Deterioration of relations with clients; JIN, Job Ineffectiveness; DIS, Disappointment; WOR, Workload; HPS, Hopelessness. ^*^p < 0.05, ^**^p < 0.01, ^***^p < 0.001.

### Mediation by trait EI and moderation by changes in workload

Regarding the mediation-moderation hypothesis, results showed that the trait EI partially mediated the relationship between every burnout dimension, which were included in the model as predictors, and hopelessness, which instead was the outcome variable (psychophysical exhaustion—BHS: B = −2.80, *p* < 0.01, 95% CI = [−3.31, −2.29]; deterioration of relations with clients—BHS: B = −3.77, *p* < 0.01, 95% CI = [−3.31,-2.29]; job ineffectiveness—BHS: B = 3.41, *p* < 0.01, 95% CI = [−3.96, −2.87]; and disappointment—BHS: B = 2.42, *p* < 0.01, 95% CI = [−2.49,-1.92]). However, the interaction between each predictor and changes in workload was non-significant (all *p*s > 0.05). Finally, all these full models accounted from 37 to 49% of the variance in hopelessness (R^2^ = 0.44, *p* < 0.001; R^2^ = 0.37, *p* < 0.001; R^2^ = 0.38, *p* < 0.001; R^2^ = 0.49, *p* < 0.001; see Table 4).

**Table 4:** Testing the mediation and moderation effect of trait Emotional Intelligence and change in workload on the relationship between Burnout dimensions and Hopelessness.

Model	Predictors	B	SE	t	95%CI
Model 1	Psychophysical Ex.	0.25^***^	0.31	8.05	[0.19; 0.31]
	TEI	−2.80^***^	0.26	10.63	[−3.31; −2.28]
	Workload	−0.58	0.68	−0.84	[−1.92; 0.76]
	Workload × psychophysical Ex.	0.03	0.03	0.35	[−0.03; −0.09]
	R^2^	0.44			
	F	110.31^***^			
Model 2	Det. relations with clients	0.10	0.03	2.64	[0.02; 0.18]
	TEI	−3.78^***^	0.25	−14.73	[−4.29; −3.28]
	Workload	−0.05	0.78	−0.06	[−1.59; 1.49]
	Workload × Det. relations with clients	0.02	0.04	0.47	[−0.06; 0.11]
	R^2^	0.37			
	F	82.09^***^			
Model 3	Job ineffectiveness	0.18^***^	0.04	4.24	[0.09; 0.27]
	TEI	−3.41^***^	0.27	−12.35	[−3.96; −2.87]
	Workload	0.38	0.66	0.57	[−0.93; 1.69]
	Workload × job ineffectiveness	0.00	0.04	−0.31	[−0.08; 0.08]
	R^2^	0.38			
	F	86.64^***^			
Model 4	Disappointment	0.30^***^	0.02	11.02	[11.10; 17.16]
	TEI	−2.41^***^	0.25	−9.59	[−2.91; −1.92]
	Workload	0.36	0.48	0.73	[−0.59; 1.31]
	Workload × disappointment	−0.00	0.02	−0.02	[−0.05; 0.05]
	R^2^	0.49			
	F	135.31^***^			

Change in workload was dummy coded such that −1 = less workload, 0 = no changes in workload, 1 = more workload. TEI, Trait emotional intelligence. ^*^p < 0.05, ^**^p < 0.01, ^***^p < 0.001.

## Discussion

The study aimed to assess the impact of the COVID-19 pandemic on the work-related stress of Italian healthcare workers involved in facing the first COVID-19 pandemic wave that, in Europe, mainly affected Italy. Our results show that northern workers have higher levels than southern workers in two burnout dimensions: psychophysical exhaustion and disappointment. This could be attributed to the increased spread of the virus in northern Italy during the first wave of the COVID-19 pandemic, with consequent greater pressure on hospitals. Furthermore, healthcare workers from the North reported significantly higher workload changes. Consistent with our findings, Swami et al. (2013) found a strong correlation between the physical fatigue dimension and changes in workload, also due to, as Karagol and Kaya (2022) found healthcare workers declared not having enough time for themselves or their own family obtained higher average scores in all dimensions of burnout and hopelessness. The authors suggest that this is due to working in departments with an extensive workload and highlight the importance to “have time for oneself or one’s own family” for mental health, as confirmed by previous studies (Hacimusalar et al., 2020; Marcia-Rodríguez et al., 2021; Tiete et al., 2021).

Regarding those who worked in COVID-19 wards, we found significant differences from those who worked in other wards only in workload changes and in the job effectiveness burnout dimension. Moreover, in contrast with our hypothesis, we found lower levels of hopelessness than those who worked in usual wards. In this regard, the literature reports some interesting data: a study conducted by Wu et al. (2020) showed that, compared with medical staff who worked on their usual wards for uninfected patients, medical staff worked on the COVID-19 front-line ward had a lower frequency of burnout, in line with another study conducted by Dinibutun (2020). According to Wu et al. (2020), a possible explanation could be that those at the front-line wards have experienced a greater closeness to the key decision-makers and were allowed to access to more timely and accurate information. On the contrary, results of other studies showing worse mental health outcomes among frontline healthcare workers during COVID-19 (Lai et al., 2020; Liu et al., 2020; Wu et al., 2020).

However, the job ineffectiveness dimension was high, and this result could be explained by the fact that physicians who were in the first line in the emergency had access to their resources to face the emergency, experiencing acute stress, but facing an unknown virus and a new disease, without standardized, has probably destabilized their sense of professional self-efficacy. These findings suggest that healthcare workers’ mental health outcomes seem to be not directly associated with the fact of working on COVID-19 front-line wards and that further studies are needed to investigate possible other variables involved.

With respect to the differences between professional profiles, nurses showed higher workload changes than physicians and, in line with our hypothesis, they scored higher in three further dimensions assessed by the LBQ: psychophysical exhaustion, deterioration with clients, and work effectiveness, as confirmed by previous studies (Lai et al., 2020; Bisesti et al., 2021; Tiete et al., 2021). These findings could be possible because nurses had more frequent contacts with patients affected by COVID-19 and were thus exposed to higher risk (Bisesti et al., 2021). Moreover, these findings also align with those reported in a previous study on the SARS epidemic (Wong et al., 2005).

Regarding gender differences, our results show that females scored higher than males only in two burnout dimensions: psychophysical exhaustion and job ineffectiveness, partially in line with previous studies in which the female gender was associated with higher levels of emotional exhaustion than the male gender (Vitale et al., 2020; Richards et al., 2021). However, it is important to note that the gender-burnout relationship is still unclear. For example, in their qualitative review of the burnout literature, Maslach (2003) observed a tendency for women to score higher on emotional exhaustion than men, whereas men tend to score higher on depersonalization than women, as confirmed by a 2010 meta-analysis (Purvanova and Muros, 2010) that highlights the fact that burnout is experienced differently by men and women, in line with gender role theory (Eagly and Wood, 1982; Eagly, 1987) which predicts that women tend to express feelings of emotional and physical fatigue (e.g., emotional exhaustion) due to the fact that they learn to display their emotions, whereas men tend to “shut off and withdraw under stress” (i.e., depersonalization) because they learn to conceal their emotions. In addition, as a possible gender roles outcome, Karagol and Kaya (2022) indicate that women’s increasing domestic workload and their less opportunity to distinguish between home and work requests, compared to men, may have led women to experience greater burnout (Karagol and Kaya, 2022). On the other hand, males score higher than females in TEI, in line with Bar-On et al. (2000) and Petrides and Furnham (2000).

Therefore, it is interesting to note that the findings in the literature are controversial. In this regard, a review by Shahzad and Bagum (2012) on gender differences in TEI reported a difference between mean scores of males and females concerning EI, with males having higher levels of TEI than females. According to the authors, a possible explanation could be that men perceive themselves as more emotionally intelligent, which could affect the results due to the self-report nature of the TEIQue. On the other hand, a gender role issue related to the social context, which defines gender roles differently, could explain the possible effects on female’s emotional development (Duckett et al., 1989; Sandhu and Mehrotra, 1999), especially if we consider the sociability subdomain in which male score slightly higher than females (Bar-On et al., 2000; Petrides and Furnham, 2000).

Regarding correlation analysis, we found that hopelessness and each burnout dimension are positively related, as confirmed by previous literature (Pompili et al., 2006; Franza et al., 2020; Akova et al., 2022; Karagol and Kaya 2022) and negatively related with trait EI. Despite many studies on burnout in healthcare workers, few studies have investigated the relationship between burnout and hopelessness during the COVID-19 pandemic (Franza et al., 2020; Akova et al., 2022; Karagol and Kaya, 2022). However, assuming that burnout may be a risk factor for the development of hopelessness and that both conditions could represent an increased risk of committing suicide, we believe that this relationship requires greater attention in order to implement psychological interventions in care setting (i.e., hospital) in the future, not only for patients and their families but also to provide support to healthcare workers and prevent the extreme consequences of burnout.

The exhaustion stage is the most severe stage of burnout and is characterized by symptoms such as chronic sadness or depression, chronic physical disorders (i.e., chronic stomach or bowel and chronic headaches), chronic mental and physical fatigue, the desire to withdraw from society, family, and friends, and also recurrent suicidal ideation (Pompili et al., 2013). In healthcare workers, hope is among the main coping strategies, able to influence people’s ability to deal with stress related to life-threatening situations (Jones-Schenk, 2020), however, “emotional exhaustion” in physicians and nurses due to providing care for a large number of patients, excessive workload and not receiving adequate financial gratifications, could promote hopelessness feelings (Karagol and Kaya, 2022).

Moreover, our results showed that trait EI could act as a protective factor against burnout levels and hopelessness, as confirmed by previous research that highlights the positive role of EI over burnout levels (Humpel and Caputi, 2001; Năstasă and Fărcaş, 2015; Vlachou et al., 2016). According to the literature, EI is associated with lower rates of burnout and higher rates of job satisfaction, self-compassion, and communication skills (De Moraes et al., 2015). Results also showed that trait EI partially mediated the relationship between every burnout dimension and hopelessness. However, full mediation was not obtained as further risk factors, including alienation and/or resources, such as self-efficacy and social support, could play a significant role in the buffering process and could potentially contribute to mediating the association between burnout and hopelessness.

The role of EI as a protective factor against burnout in a nurse’s sample during the COVID-19 pandemic has already been observed by Soto-Rubio et al. (2020). The authors highlight the importance of nurses’ abilities to perceive and regulate their emotions while being aware of and empathizing with others’ emotions (Soto-Rubio et al., 2020). Several studies conducted during the COVID-19 pandemic took into account EI as a protective factor against psychosocial risks such as burnout and psychosomatic complaints and a favorable effect on job satisfaction (Soto-Rubio et al., 2020; Lisigurski et al., 2021; Richards et al., 2021; Zeb et al., 2021; Shariatpanahi et al., 2022), but to our knowledge, no study on the relationship between trait EI, burnout, and hopelessness has been conducted yet.

Finally, our results in the mediation and moderation model partially confirmed our hypothesis: TEI partially mediates the relationship between burnout and hopelessness, and this could reduce the risk of the onset of psychopathology. Being flexible and willing to adapt to new conditions, knowing how to recognize, regulate, express, and communicate emotions, being successful, self-confident, talented in networking, and capable of coping with pressure and regulate stress act as a buffer to burnout consequences, reducing the risk of developing negative cognitive patterns in which the negative expectation toward the future prevails. Hopelessness is, in fact, characterized by the tendency to attribute negative life events to internal, stable, generalizable factors that could lead to suicidal ideation. For these reasons, there seems to be a clear need for psychological interventions aimed at improving the emotional awareness of healthcare workers. Working on these abilities, people who work in a high-risk context could better manage emergencies and stress. The COVID-19 pandemic, in its dramatic nature, has highlighted the importance of the role of the psychologist within hospitals. Interventions such as Balint group (Huang et al., 2020) and mental health services (Sultana et al., 2020) have already proven to be effective ways to relieve burnout and improve the quality of work life for healthcare workers.

In Italy, during the COVID-19 pandemic, the National Council of the Order of Psychologists (CNOP) sought to provide emergency and structural responses to the growing need for psychological support for healthcare workers, such as the creation of task forces of psychologists in healthcare facilities.^3^

However, in this study, the mediation of TEI in the burnout-hopelessness relationship partially explains the protective role of individual factors against burnout, even in emergencies such as the first COVID-19 pandemic wave.

Regarding the possible moderator role of changes in workload in the burnout-hopelessness relationship, our analysis does not support this hypothesis. However, this finding highlight how individual factors have more influence on suicide risk, even in high-risk individuals such as healthcare workers with burnout.

Individuals with high levels of hopelessness believe that nothing will turn out in their favor, that they will never be successful in life, that their important goals will not be achieved, and that their problems will not be solved, regardless of the actual situation.

## Limitations

Our study suffers from some limitations. First of all, the cross-sectional design does not allow us to grasp the effect of the changes in the variables over time. Moreover, the use of self-reports only may be associated with common method bias. Moreover, even though recruitment procedures (i.e., snowball sampling method through social media, emails, and the university’s website) allowed us to reach as many voluntary participants as possible during the lockdown, the sample composition could be biased due to the fact that online recruitment procedures tend to select individuals who are more active on both the internet and social media platforms. Finally, some other variables (personal and/or situational) that should influence the results could be excluded from the analysis and not controlled.

## Conclusion

Although much attention has been paid to the effects of the COVID-19 pandemic on burnout and mental health in healthcare workers, little has been carried out so far to understand the underlying specific mediation and moderation mechanisms. To our knowledge, this is the first study to examine the role of trait EI in burnout syndrome in healthcare workers during the COVID-19 pandemic. The model we tested could more effectively grasp the complexity of the conditions through which a significant specific work-related stress can affect healthcare workers’ mental health. The model, in fact, included protective (TEI) and risk (burnout) psychological factors that could have influenced psychopathological risks such as hopelessness and suicide risk. Our findings indicated that trait EI partially mediated the relationship between burnout and hopelessness; these effects were gender- and age-range-independent. This study identifies a pathway (i.e., via trait EI) that may help better understand the mechanism by which the extreme consequence of burnout syndrome, such as suicide risk, could be limited. Our findings support the need to integrate considerations on both psychological risk and protective factors into COVID-19 care, including the monitoring of psychological symptoms and social needs, especially among healthcare workers. Therefore, we believe that health promotion policies must take into account both contextual and personal variables, in order to plan in advance intervention for the high-risk group.

## Data availability statement

The data that support the findings of this study are available from the corresponding author upon reasonable request.

## Ethics statement

The studies involving human participants were reviewed and approved by Bioethics Committee of the University of Palermo (no. 7/2020). The patients/participants provided their written informed consent to participate in this study.

## Author contributions

ME, SG, ET, and FA: conceptualization. ME, SG, FA, and LP: methodology. MR, VS, MF, and GM: data collection and data curation. MP: data analysis. ME, MP, VS, and MR: writing the original draft manuscript. ME, MP, and FA: review and editing. ME, SG, FA, and ET: supervision. All authors contributed to the article and approved the submitted version.

## Conflict of interest

The authors declare that the research was conducted in the absence of any commercial or financial relationships that could be construed as a potential conflict of interest.

## Publisher’s note

All claims expressed in this article are solely those of the authors and do not necessarily represent those of their affiliated organizations, or those of the publisher, the editors and the reviewers. Any product that may be evaluated in this article, or claim that may be made by its manufacturer, is not guaranteed or endorsed by the publisher.

## Footnotes

^1^ https://portale.fnomceo.it/

^2^ https://www.epicentro.iss.it/

^3^ https://www.psy.it/covid-19-e-gestione-dello-stress-al-via-liniziativa-per-il-supporto-psicologico-del-personale-sanitario/
